# Molecules and Prostaglandins Related to Embryo Tolerance

**DOI:** 10.3389/fimmu.2020.555414

**Published:** 2020-11-19

**Authors:** Gabriel Mayoral Andrade, Gabriela Vásquez Martínez, Laura Pérez-Campos Mayoral, María Teresa Hernández-Huerta, Edgar Zenteno, Eduardo Pérez-Campos Mayoral, Margarito Martínez Cruz, Ruth Martínez Cruz, Carlos Alberto Matias-Cervantes, Noemi Meraz Cruz, Carlos Romero Díaz, Eli Cruz-Parada, Eduardo Pérez-Campos

**Affiliations:** ^1^ Research Centre Medicine National Autonomous University of Mexico-Benito Juárez Autonomous University of Oaxaca (UNAM-UABJO), Faculty of Medicine, Benito Juárez Autonomous University of Oaxaca, Oaxaca, Mexico; ^2^ Biochemistry and Immunology Unit, National Technological of Mexico/ITOaxaca, Oaxaca, Mexico; ^3^ CONACyT—Faculty of Medicine, Benito Juárez Autonomous University of Oaxaca, Oaxaca, Mexico; ^4^ Department of Biochemistry, School of Medicine, UNAM, Mexico City, México; ^5^ School of Medicine, Branch at National Institute of Genomic Medicine, Mexico City, Mexico

**Keywords:** prostaglandins, PGE2, platelets, polymorphonuclear leukocyte, group 2 innate lymphoid cells, embryo tolerance

## Abstract

It is generally understood that the entry of semen into the female reproductive tract provokes molecular and cellular changes facilitating conception and pregnancy. We show a broader picture of the participation of prostaglandins in the fertilization, implantation and maintenance of the embryo. A large number of cells and molecules are related to signaling networks, which regulate tolerance to implantation and maintenance of the embryo and fetus. In this work, many of those cells and molecules are analyzed. We focus on platelets, polymorphonuclear leukocytes, and group 2 innate lymphoid cells involved in embryo tolerance in order to have a wider view of how prostaglandins participate. The combination of platelets and neutrophil extracellular traps (Nets), uterine innate lymphoid cells (uILC), Treg cells, NK cells, and sex hormones have an important function in immunological tolerance. In both animals and humans, the functions of these cells can be regulated by prostaglandins and soluble factors in seminal plasma to achieve an immunological balance, which maintains fetal-maternal tolerance. Prostaglandins, such as PGI2 and PGE2, play an important role in the suppression of the previously mentioned cells. PGI2 inhibits platelet aggregation, in addition to IL-5 and IL-13 expression in ILC2, and PGE2 inhibits some neutrophil functions, such as chemotaxis and migration processes, leukotriene B4 (LTB4) biosynthesis, ROS production, and the formation of extracellular traps, which could help prevent trophoblast injury and fetal loss. The implications are related to fertility in female when seminal fluid is deposited in the vagina or uterus.

## Introduction

Prostaglandins (PGs) belong to a subclass of eicosanoids known as prostanoids, these are comprised of C20 atoms, including a cyclopentane ring. PGs are hormone-like chemical messengers which act as autacoids (1) through prostanoid receptors (G protein-coupled receptors) and their variants or isoforms such as E_1-4_, DP_1-2_, FP, TP, and IP (1, 2). The main precursor of eicosanoids is arachidonic acid (AA), this is released by the action of phospholipases A2 (PLA2) and C (PLC) (3), AA is then converted into different metabolites through the COX, LOX, and CYP450 pathways (4). The importance of prostaglandins becomes evident when ovulation and fertilization are affected, e.g., as cyclooxygenase (COX) is inhibited by aspirin or indomethacin (5).

PGs have a significant role in maternal immune tolerance and the conception process. We consider prostaglandins in seminal fluid as key in modulating responses in different types of cells participating in fetal-maternal tolerance.

The balance of the immune response in maintaining fetal-maternal tolerance is due to a complex network of soluble molecules and cells, such as macrophages, and dendric, decidual, and NK cells. In **Table 1**, cells and biological processes are summarized. Moreover, many molecules are released by these cells and have a fundamental role in the tolerance process. **Table 2** summarizes the most important of these.

**Table 1:** Cells related to maternal-fetal tolerance and implantation.

	Cells	Biological process	Molecules related	Prostaglandins related	Authors
**Dendritic cells**	ILT4^+^ Dendritic cells (DCs)	Induction of Foxp3^+^ Treg cells. DCs suppress T-cell activity, induce T helper cell anergy and inhibit the differentiation of cytotoxic T cells.	IL-10		Liu et al. (6)
	Tolerogenic dendritic cells (tol-DCs)	Present the antigen to Th0 cells, which become activated, proliferate and differentiate into peripherally derived Tregs (pTregs).		PGE2–EP4 receptor signaling inhibits IL-12 and promotes IL-23 production. PGE2 regulates IL-10 production.	Flórez-Grau et al. (7) Robertson et al. (8)
**Macrophages**	M1 macrophages	Skew T cell responses to a TH1 mediated immune response.	IL-12, IL-23, ROS	PGE2 is essential to corpus luteum formation by stimulating macrophages to induce angiogenesis through EP2/EP4.	Brown et al. (9) Liu et al. (10)
	M2 macrophages	Promote TH2 or antibody mediated immune responses.	IL-10, TGF-β	PGD2, PGF2α, and PGE2 contribute to differentiation toward M2‐like macrophages	Brown et al. (9) Magatti et al. (11)
**NK cells**	Uterine NK cells (uNK)	Respond to fetal MHC class I molecules. Stimulate fetal growth. Regulate decidual blood vessel remodeling.	IFN-γ, growth-promoting factors		Sojka et al. (12) Fu et al. (13)
	Endometrial NK cells (eNK)	Inactive cells (before IL-15 activation) that are present in the endometrium before conception and pregnancy.	IP-10 or IFN-γ		Yang et al. (14) Manaster et al. (15)
	Decidual NK cells (dNK) (CD56^bright^CD16^-^)	Widen maternal blood vessels and promote fetal growth. Interact with resident myeloid cells and participate in the induction of regulatory T cells	IL-24, Angiopoietin 1 and 2 (Ang 1, Ang 2), vascular endothelial growth factor C (VEGF‐C), TGF‐β1, SDF-1, pleiotrophin, osteoglycin, IL‐8, protein‐10.	Suppression of their activity has been observed in humans and mice by PGE2.	Yang et al. (14) Yu et al. (16)
**Decidual cells**	Decidual stromal cells (DSCs)	Differentiation and development of dNK during decidualization. Induce the downregulation of activating NK receptors and inhibit NK cell proliferation, cytotoxicity, and IFN-γ production.	IL-24, TGF-β	The DSC-induced inhibition is primarily mediate by PGE2.	Yang et al. (14) Sojka et al. (12) Vacca et al. (17) Croxatto et al. (18)
	Decidual ILC3 (NCR^+^NCR^−^)	Establish physical and functional interactions with neutrophils and produce factors for pregnancy induction/maintenance and promotion of the early inflammatory phase.	IL-8, IL-22, GM-CSF, TNF, IL-17		Vacca et al. (17)
	Decidual Tregs	Express CD25, CTLA4, and PD-L1, which are hallmark mediators of Treg suppression. Downregulate DC costimulatory molecules CD80 and CD86 needed for T effector (Teff) activation.	IL-10, TGF-β		Robertson et al. (8)
	Decidual T cells	Proliferate in response to fetal tissue. Elevated expression of proteins associated with the response to interferon signaling.	IL-4, IL-10, IFN-γ, leukaemia inhibitory factor and colony-stimulating factor 1 (M-CSF).		Ernerudh et al. (19) Powell et al. (20)
	Decidual myeloid cells (dCD14^+^)	Induce Treg, dNK and dCD14^+^ cells resulting in the production of IFN-γ.	TGF-β, indoleamine 2,3-dioxygenase (IDO).		Vacca et al. (17)
	Decidual CD4^+^EM cells	Increase expression of the immune inhibitory checkpoint receptors PD-1, Tim-3, cytotoxic T lymphocyte antigen 4 (CTLA-4), and lymphocyte activation gene 3 (LAG-3).	IFN-γ, IL-4		Kieffer et al. (21)
	Decidual CD8^+^EM cells (CD45RA^−^CCR7^−^)	The interaction with trophoblasts induces the upregulation of Tim-3 and PD-1. Trophoblasts may induce tolerance in CD8^+^ EM cells in the decidua. Reduced expression of perforin and granzyme B.	IFN-γ, IL-4	PGE2 is an important modulator of CD8 membrane expression in human lymphocytes.	Kieffer et al. (21) Tilburgs et al. (22) Ouellette et al. (23)
**T Cells**	Tregs (CD4^+^CD25^+^FOXP3^+^)	Inhibit the activation and function of Th1 and Th17 cells and control inflammation. Control IL-15 release from DCs and suppress uNK cytolytic activity.	TGF-β, IL-10, Heme oxygenases‐1(HO-1)	PGE2 promotes the development of regulatory T cells.	Robertson et al. (8) Erkers et al. (24)
	T helper 3 cells (Th3 cells)	Induce the mucosal environment that is intrinsically rich in TGF-b, IL-10, and IL-4. Vasoactive intestinal peptide (VIP) modulates to toward a tolerogenic profile.	VIP, TGF-β		Ernerudh et al. (19) Grasso et al. (25)
	Th17 cells	Express higher levels of T cell immunoglobulin- and mucin-domain-containing molecule-3 (Tim-3) and programme death-1 (PD-1) inducing inflammation. Regulating trophoblast function.	IL-10		Ahmadi et al. (26) Wang et al. (27)
**Endometrial and umbilical cells**	Endometrial stromal cells (ESCs)	In humans, the so-called “decidualization window” transforms endometrial stromal cells into larger round decidual cells. This phenomenon is largely dependent on hemodynamic forces, progesterone, and prostacyclin.	IL-15	PGE2 can stimulate IL-15 expression and release by ESCs	Dunn et al. (28) De Clercq et al. (29) Gnecco et al. (30)
	Human Umbilical Vein Endothelial Cells (HUVECs)	Possess immune-regulating properties and are one of the first fetal cells to make contact with foreign maternal immune cells. Also, increase the Treg cell population.	TGF-β		Oettel et al. (31)

**Table 2:** Principal soluble molecules acting in implantation (apposition/adhesion/invasion) to maintain fetal-maternal tolerance.

Effects	Soluble molecule	Biological process	Steroid hormones and related molecules	Author
Attachment and implantation	Oestrogen	Regulation of oestrogen receptors β/IL-24 (ERβ/IL‐24) signal pathways. Induces the recruitment of macrophages and DCs.	Promotes the conversion of peripheral Tregs in secondary lymphoid organs. Prolongs the survivals of H-Y skin grafts by the expansion of Tregs, suppression of CD3(+) CD8(+) effector T-cells and immune shifts toward Th2 cytokines.	Padmanabhan et al. (32) Vrtačnik et al. (33) Lin et al. (34)
	17β-oestradiol (E2)	Promotes uterine blood flow, myometrial growth stimulates breast growth and later promotes cervical softening and expression of myometrial receptors. Expansion and activation of monocytic-myeloid-derived suppressor cells (M‐MDSCs) through signal transducer and activator of transcription (STAT)‐3.	E2‐treated MDSCs have a stronger capability in suppressing T cell responses. 17β-oestradiol, FSH, oxytocin, and arachidonic acid (AA) induce receptors and enzymes through the synthetic pathway for PGE2.	Rahimipour et al. (35) Pan et al. (36) Falchi and Scaramuzzi, (37)
	Progesterone (P4)	Stimulates the activity of some specific enzyme matrix metalloproteinases and adhesion molecules. Inhibits antibody production and suppresses T-cell activation and cytotoxicity and modifies the activity of natural killer cells; influences B cells and induces secretion of protective asymmetric antibodies.	Progesterone-induced blocking factor (PIBF) mediates the immunomodulatory effects of progesterone. Consumption of IL-4 increases and the number of cells undergoing apoptosis. Increases secretion of IL-10, IL-27, causes increased secretion of IL-13 and decreased secretion of IL-23 by the monocyte-derived dendritic cells. Upregulates macrophage-colony-stimulating factor (M-CSF) and downregulates granulocyte-macrophage colony-stimulating factor (GM-CSF). Progesterone and prostaglandin E have synergistic inhibition effects on T-cell mitogenesis.	Rahimipour et al. (35) Kyurkchiev et al. (38) Svensson et al. (39) Fujisaki et al. (40)
	Chorionic gonadotropin (CG)	hCG is comprised of 4 molecules, one produced by villous syncytiotrophoblastic cells, another hyperglycosylated hCG produced by cytotrophoblast cells, the free beta subunit, and hCG produced by anterior pituitary gonadotropic cells. Stimulates P4 production by the corpus luteum, facilitating trophoblast invasion, and promoting angiogenesis.	It is a pleiotropic molecule that mediates implantation. Upregulation of indoleamine 2,3-dioxygenase activity of dendritic cells. hCG may have a biological role in the regulation of PG (PGE and 6-keto-PGF1) synthesis in trophoblasts. In particular, the hyperglycosylated form stimulates implantation through the invasion of cytotrophoblast cells.	Cole, 2020. (41). Szmidt et al. (42) Bansal et al. (43) Schumacher et al. (44) North et al. (45)
	Neuropeptide kisspeptin (KP)	Kisspeptins participate in reproduction. Regulates trophoblast cell invasion alongside tumor necrosis factor α.	KP is a regulator of Gonadotropin (GnRH) secretion and stimulates LH secretion and LH pulse frequency. KP-10 moderates trophoblast invasion and regulating implantation.	Mumtaz et al. (46) Francis et al. (47) Skorupskaite et al. (48) Pinilla et al. (49)
	Platelet-Activating Factor (PAF)	Platelet-activating factor is an acetylated Glycerophospholipid, releasing histamine from platelets, which increase vascular permeability.	PAF is related to processes of ovulation, implantation and parturition, and is regulated by ovarian steroid hormones. PAF is associated with sperm motility, acrosome reaction, and fertilization.	Harper, 1989. (50) Tieman, 2008. (51) Roudebush, 2001. (52)
Cytokine mediators of implantation and decidualization	IL-6	IL-6 is a cytokine with functions in immunity, metabolism and tissue regeneration. It is produced in the endometrial epithelium and stromal cells during implantation.	Variation in the expression of pro-inflammatory cytokines such as IL-6, CSF-1, macrophage colony-stimulating factor (CSF-1), granulocyte-macrophage colony-stimulating factor (GM-CSF), interleukin 1-alpha, interleukin 1-beta, and tumor necrosis factor-alpha (TNF alpha) has been reported in the uterus immediately after mating in mice. Changes in the bioavailability of IL-6 are important for pregnancy. The increase of IL6 is related to unexplained infertility, recurrent miscarriage, preeclampsia and preterm delivery and inhibition of the generation of CD4 + regulatory T cells in pregnancy tolerance. Local IL-6 insufficiency could also contribute to recurrent spontaneous abortion. IL6 activate cathepsin S (CTSS) in dendritic cells, in decidualized endometrial stromal cells, this process is regulated by cystatins CST7 and CST3.	De et al. (53) Ochoa-Bernal et al. (54) Cork et al. (55) Prins et al. (56) Baston-Buest et al. (57)
	Leukaemia inhibitory factor (LIF)	It is a member of the interleukin-6 family of cytokines. Upregulation of poFUT1, promotes trophoblast cell migration, invasion and differentiation at the fetal-maternal interface through activating the Janus kinase/signal transducers and fetal transcription (JAK/STAT) and a *mitogen-activated protein kinase* (MAPK) signaling pathway.	Urokinase-type plasminogen activator receptor (uPAR) is upregulated by LIF, also it is mediated by phosphoinositide-3-kinase–protein kinase B/Akt (PI3K/AKT) signaling pathway. LIF participates in placentation by up-regulating PGE2 production and PGE2 receptor expression.	Szmidt et al. (42) Liu et al. (58) Zheng et al. (59) Horita et al. (60)
	IL-1	Acts on blastocysts, syncytiotrophoblasts and endometrial glands.	Stimulates endometrial secretion of endometrial leukaemia inhibitory factor (LIF), prostaglandin E2, and integrin β3 subunit expression.	Viganò et al. (61) Hambartsoumian, 1998. (62) Fouladi-Nashta et al. (63)
	IL-11	IL-11 regulates endometrial epithelial cell increasing adhesion to fibronectin and collagen IV, similar to IL-6.	IL-11 decreases TNFα in a dose-dependent way in epithelial and stromal cells, in endometria, through gp130. IL-11 production is maximal during decidualization, its production depends on steroid hormones, relaxin and PGE2.	Cork et al. (55) Marwood et al. (64) von Rango et al. (65)
	IL-15	Promotes the differentiation of the local eNK cells toward dNK cells.	Decidual NK cells secrete cytokines and angiogenic factors to placental vascular remodeling and differentiation. IFN-γ, IP-10, vascular endothelial growth factor (VEGF), Placenta growth factor (PlGF). Suppression of IL-15-activated NK cell is mediated by PGE (2).	Manaster et al. (15) Kopcow and Karumanchi, 2007. (66) Joshi et al. (67)
	IL-24	Regulates the function of eNK and pNK through the Janus kinase (JAK)/STAT3 pathway.	Contributes in differentiation to CD56^bright^CD16^−^dNK with low cytotoxic activity, high immunomodulation and angiogenic activity by inhibiting CD16, Granzyme B and perforin, IFN‐γ, upregulating KIR2DL1, KIR3DL, TGF‐β, IL‐10, and IL‐8.	Yang et al. (14)
	Cytokine-like protein 1 (Cytl1)	Regulation of embryo implantation. It is an ovarian hormone-dependent protein expressed in the endometrium that stimulates the secretion of LIF and heparin-binding epidermal growth factor (HB-EGF). Induces endometrial cell proliferation.	Releases LIF, HB-EGF, and IL-1, in decidualization.	Ai et al. (68) Wang et al. (69) Moghani-Ghoroghi et al. (70)
Implantation and decidualization	Cellular Adhesion Molecules (CAMs)	Adhesion molecules include integrins, cadherins, selectins, and the immunoglobulin superfamily.	Numerous integrins interact with the trophoblast, especially the αVβ3, with its ligand osteopontin. HOXA 10 and IL-1 regulated β3 subunit expression in the receptive endometrium. The absence of L-selectin and its Meca-79 ligand is associated with recurrent implantation failure (RIF), also, a significant reduction of HOXA-10 and E-cadherin in recurrent implantation failure (RIF) and recurrent miscarriage (RM). ICAM-1, VCAM-1, NCAM, CD44, and CD49d provide interaction between the embryo and maternal cells.	Achache and Revel, 2006. (71) Foulk et al. (72) Yang et al. (73) Lu et al. (74)
	Melatonin	Melatonin is an indoleamine acting as an antioxidant, free radical scavenger, and it promotes embryo development in different species	A positive feedback loop among p53, p38, and p21 inhibiting mucin 1 and activating LIF is realized by melatonin signaling, which improves adhesion proteins, present at the membrane level on endometrial cells and the blastocyst, in the pre-implantation stage. Melatonin is associated with the inhibition of prostaglandin synthesis.	Carlomagno et al. (75) Voiculescu et al. (76) Gimeno et al. (77)
	Calcitonin (CT)	It is a peptide hormone which regulates calcium homeostasis	Promotes endometrial receptivity and embryo implantation.	Xiong et al. (78) Xiong et al. (79)
	Platelet-derived growth factor (PDGF-BB)	Decidualized endometrial stromal cells migrate upon exposure to PDGF-BB.	Involvement of ERK1/2 and PI3K/Akt signaling in endometrial stromal cell chemotaxis. Both epidermal growth factor (EGF) and platelet-derived growth factor (PDGF) participate in implantation in the first days of gestation.	Schwenke et al. (80) Jaber and Kan, 1998. (81)
	Platelet-derived growth factor (PDGF-AA)	Secreted by the trophoblast cell line AC-1M88 and by first trimester villous explants. Trigger endometrial stromal cell chemotaxis.	Participates in attracting decidualized endometrial stromal cells to the implantation site. Modulates early post-implantation.	Schwenke et al. (80) Haimovici and Anderson, 1993. (82)
	Tissue inhibitor of MMP (TIMP)	Endogenous inhibitor of MMP activity in tissues.	Inhibits trophoblast invasion. Decidual cell production. TIMP-2 attenuates the proteolysis of IGFBP-1 by MMP-3.	Liu et al. (58) Coppock et al. (83)
	Heparin-binding epidermal growth factor (HB-EGF)	HB-EGF has a function in implantation, decidualization and placenta development. Promotes differentiation of trophoblast cells to the invasive phenotype. Stimulates the migration of decidualized endometrial stromal cells.	Endometrial stromal cells with HB-EGF increase the level of the tetraspanin CD82, a metastasis suppressor found in decidual cells at the implantation site. A decreased level of HB-EGF is related to pregnancy complications.	Schwenke et al. (80) González et al. (84) Ozbilgin et al. (85)
	Lipoxins	These are derived from arachidonic acid, an ω-6 fatty acid. They exert their anti-inflammatory effects through binding to high-affinity G protein-coupled lipoxin receptors.	Lipoxins, calcitonin, leukaemia inhibitory factor, and homeobox A10 are essential in implantation. Lipoxin A4 is regulated by human chorionic gonadotrophin (hCG) during early pregnancy and it has anti-inflammatory activity in human endometrium and decidua tissue.	Xiong et al. (79) Macdonald et al. (86)
	Complement components and their receptors (C1q, gC1q, α4β1 integrin)	It is produced at the fetal-maternal interface by macrophages, decidual endothelial cells and invading trophoblasts.	Synthesis of C1q by decidual endothelial cells is crucial for the replacement by endovascular trophoblasts. Surfactant proteins SP-A and SP-D play a role in implantation, trophoblast invasion and placental development.	Agostinis et al. (87) Madhukran et al. (88)
	Protein O-fructosyltransferase 1 (poFUT1)	Favors trophoblast cell migration and invasion at the fetal-maternal interface.	Increases Tissue inhibitors of metalloproteinases 1 and 2 (TIMP-1, TIMP-2) expression further inhibited MMP-2 activity. Activates MAPK and PI3K/Akt signaling pathways.	Liu et al. (58) Liu et al. (89)
	Matrix metalloproteinase (MMP-2) -2	Implicated in the remodeling of the extracellular matrix (ECM) during the trophoblast invasion process.	Synthesis and degradation of the extracellular matrix under physiological and pathological conditions. It is capable of degrading collagen. During the implantation process, matrix metalloproteinase (MMP)/insulin-like growth factor binding protein-1 (IGFBP-1) activity is stimulated by leukaemia inhibitory factor (LIF) and colony-stimulating factor (CSF).	Liu et al. (58) Ortega et al. (90) Herrler et al. (91)
	Gonadotropin-releasing hormone type II (GnRH-II) agonist	Promotes cell motility of human decidual endometrial stromal cells through the GnRH-IR by phosphorylation of ERK1/2 and JNK in decidual endometrial stromal cells.	Increased expression and proteolytic activity of matrix metalloproteinase-2 and -9 (MMP-2, MMP-9) is due to GnRH-II	Wu et al. (92)
Immune tolerance	Human leukocyte antigen G (HLA-G)	Promotes proliferation and cytokine production by uNK cells.	Secretion of growth-promoting factors essential for fetal development by uNK cells. Levels of sHLA-G ≥ 2 U/ml in embryos which were selected for transfer after IVF based on culture media gave a 65% pregnancy rate compared with low levels of sHLA-G. The HLA-G -725 promoter polymorphism has a high risk for recurrent miscarriage.	Sojka et al. (12) Roussev and Coulam, (93)
	Soluble MHC class I (sMHC‐I)	sMHC-I induces apoptosis by stimulating expression of CD95-L and regulates the Fas/FasL system.	sHLAs downregulates T-cell responses.	Bakela and Athanassakis, (94) Zavazava and Krönke, 1996. (95)
	Soluble MHC class II (sMHC‐II)	It has important immunoregulatory properties, stimulates proliferation of CD25− CD4+, CD25+ CD8+ and CD25+ CD4+ cell, as well as inhibits CD25− CD8+ cells.	sMHC‐II decreases IL‐2, increases IL‐10, and inhibits phosphorylation of ZAP‐70, particularly LAT proteins in the pathways of TCR signaling in CD4+ cells.	Bakela and Athanassakis, 2018. (95) Athanassakis and Vassiliadis, 2003. (96)
	Pregnancy specific beta-1-glycoprotein 9 (PSG9)	Binds to the 250-residue latency-associated peptide (LAP) and activates the latent form of TGF-β1.	Induces the secretion of TGF-β1 from macrophages. Induces the differentiation of FoxP3+ regulatory T-cells from naive T-cells. PSG participates in immune tolerance in pregnancy by suppressing the CD16/56 expression by NK-cells and enhancing the CD16/56 expression by NKT-cells.	Jones et al. (97) Zamorina and Raev, 2015. (98)
	Alpha-fetoprotein (AFP)	It is released by trophoblasts during pregnancy. Acts as a fetal transport protein. Influences fetal-maternal immunologic relationships during the first trimester and helps to protect the foetus against attacks by the maternal immune system.	Suppresses the production of TNFα and IL-1β. Controls the production of HLA-G and the Ia antigen, it stimulates the growth of trophoblasts containing paternal H2 antigens. Inhibits macrophage expression of Ia antigens. AFP is capable of driving B cells into apoptosis to avoid maternal B cells in order to reach the foetus.	Schumacher et al. (44) Lafuste et al. (99) Lu et al. (100) Fettke et al. (101)
	Indoleamine-2,3-dioxygenase (IDO)	IDO is involved in tolerance	IDO activity promotes tolerance due to the conversion of mature dendritic cells (DCs) into tolerogenic antigen-presenting cells (APCs) that suppress effector T cells (Teff) and promote regulatory T cells (Tregs). Factors which are expressed by Human amniotic membrane-derived mesenchymal stem cells (hAM-MSCs) including hepatocyte growth factor (HGF), TGF-β, prostaglandin E2 (PGE2), and indoleamine 2,3 dioxygenase (IDO) have immunomodulatory effects.	Mellor et al. (102) Mellor et al. (103) Kang et al. (104)
	Preimplantation Factor (PIF)	It is a fifteen amino acid linear peptide secreted by embryos two-cell, four-cell and six-cell stages in mice, in humans and bovines, respectively	PIF promotes immunological tolerance due to increasing the expression of HLA-G, -C, -E, and -F slightly. It also potentiates the effect of the endogenous steroid and promotes the secretion of Th1/Th2 cytokines.	Hakam et al. (105) Zare et al. (106)

The molecules are released through macro-, micro-, and nanovesicles, including exosomes from placenta cells, syncytiotrophoblasts, denudated syncytiotrophoblasts, and extravillous trophoblasts. All are part of the complex intercommunication between the foetus and the mother. These vesicles transport immunomodulatory proteins such as Fas ligand, TRAIL, CD274, CD276, HLA-G5, Syncytin-1, hCG, glycodelin, galectin-1 (107), which may maintain fetal-maternal tolerance, and may even be related to recurrent early miscarriage (108).

The accumulated evidence indicates that when sexual intercourse occurs and seminal fluid is deposited in the female reproductive tract, the prostaglandins in the seminal fluid, i.e., PGE2, PGE1, PGE3, and PGF2 (109), initiate a signaling cascade toward the woman’s innate immune cells. The cells mentioned in **Table 1**, such as platelets, polymorphonuclear leukocytes, and Group 2 innate lymphoid cells participate in the physiological mechanisms in embryo tolerance and implantation, allowing successful fertilization.

## Preimplantation, Implantation, and Decidualization

Implantation begins by apposition and adhesion of the embryo to the luminal epithelium of the endometrium. Following its invasion toward the stromal bed, the union of the embryo to the luminal epithelium transforms the underlying stromal fibroblasts into secretory cells of the epithelioid type, or decidualization (110). Through different molecules such as IL-1β, steroid hormones, insulin-like growth-factor-binding protein-1 (IGFBP-1) and prostaglandin-endoperoxide synthase-2 (PTGS-2), the decidualized cells regulate this stage with the invasion of embryos, and the formation of the placenta (110).

Prostaglandins participate in each stage of the interaction of the embryo with the endometrium, for example in preimplantation, implantation (apposition, adhesion/attachment, invasion/penetration) and decidualization; as well as affecting many other cells and molecules. PGs have a complex role in each of these stages, e.g., the essential role of prostaglandin E2 (PGE2) in the oocyte is to enhance the cumulus expansion in ovulation for sperm penetration, to regulate extracellular matrices disassembly (111), and also, importantly, to participate during transport and embryo implantation (112).

## Prostaglandin Signaling by Seminal Fluid and Fertilization

Preceding evidence shows that sperm induces immunosuppression against hapten-modified self and alloantigens, including cytotoxic T-cell in mice responses (113). Also, seminal plasma contains high concentrations of prostaglandins, key molecules in the regulation of sexual intercourse signaling (114). The female immune response tolerates seminal plasma and supplies cytokines and prostaglandins, which are synthesized in the male accessory glands. In addition, it causes molecular and cellular changes in the endometrium. This facilitates the development and implantation of the embryo when prostaglandins, cytokines and hormones bind to receptors in target cells in the cervix and uterus (115).

The prostaglandins present in seminal fluid have a role in immune modulation. They regulate the pathways that may exacerbate inflammation in the female reproductive tract during physiological processes such as ovulation, implantation, and parturition (116), e.g., ejaculation or the spermatozoa induce an inflammatory response in the endometrium in the preimplantation period after mating, in which IL-1 (alpha and beta), and TNF-alpha participate (117).

Seminal plasma derived from the male accessory sex glands performs a fundamental function in fertilization in animals. The components of seminal plasma participate in the transport and survival of viable sperm and the elimination of non-viable sperm from the uterus (118). In the quail species, the cloacal gland produces prostaglandin F2α (PGF2α), which contributes to successful fertilization and acts as a natural mechanism for the protection of sperm from rejection or death by the female reproductive tract (119). Seminal fluid factors exert significant effects on the female reproductive tract, as shown by Shahnazi et al. (120). Also, in the uterine tissues of mice that were paired with mice without seminal vesicles, implantation rates, enzyme cytosolic PGE synthase (cPGES), microsomal PGE synthase (mPGES) and receptors EP2 and EP4 involved in the signaling pathway of PGE2, were all significantly low (120). In addition, 19-hydroxy PGE and 19-hydroxy PGF are regulators of sperm motility, and its effects may be mediated by the content of ATP in sperm (121). Prostaglandins such as PGE-1 are potent stimulators of adenylate cyclase in various cellular systems (122). An increase in adenylate cyclase activity and subsequent entry into cAMP levels may also be involved. PGs stimulate the fertilization capacity of human sperm by facilitating the transport of calcium through their plasma membrane (123).

The amplification of effects by microparticles from epididymal fluid (epididymosomes) and prostasomes could lead to the activation of many genes and the expression of related molecules, as reported in humans and mice, some species of cows, pigs and sheep (123, 124). More specifically, signaling may affect the enzymes of the cyclooxygenase pathway and other molecules related to the metabolism of arachidonic acid, e.g., Cytochrome P450 in blastocyst implantation (125), and prostaglandin D2 in the maintenance of pregnancy through Th1/Th2 and T-cytotoxic (Tc) 2 cells balance (126, 127).

The change induced by seminal plasma in a porcine uterus makes conception and pregnancy possible (128), it also reduces embryonic mortality in pigs and other livestock (129). In addition, seminal plasma possesses potent immunosuppressive activity caused by immune-deviating soluble factors, inducing tolerance, with molecules, such as Transforming growth factor-β (TGFB) and prostaglandin E (PGE).

## Effects of Prostaglandins and Related Molecules on Innate Immunity and Female Reproductive Tract Cells

Cells of the innate immune response are modulated by prostaglandins (130), among them, are the following:

1. M1 macrophages (Mø1) which produce proinflammatory cytokines (TNFα, IL-6, IL-12, IL-23, and IL-1β), M2 macrophages(Mø2) which produce IL-10 and TGFβ (transforming growth factor β) and have anti-inflammatory and immune down-regulating properties. Both are regulated by prostaglandins in pregnancy (9) (**Table 1**).
2. Dendritic cells (DCs) have several subclasses, e.g., CD103^+^, myeloid, plasmacytoid, the latter are related to the production of high IFNα levels. In infertile patients with endometriosis, CD4^+^, CD25^+^, and CD103^+^ dendritic cells are increased in peritoneal fluid (131), dendritic cells CD103^+^ have a relevant role in implantation (132); in addition, CD103^+^ dendritic cells are regulated by prostaglandin D2 in different disorders (133).
3. Endothelial cells have innate and immune tolerogenic function (134). In patients with preeclampsia (PE), in the presence of vascular endothelial growth factor (VEGF), these cells increase levels of prostacyclin (135). In the pathogenesis of PE, VEGF (VEGF-A) participates in the proliferation, migration and angiogenesis of endothelial cells, and works through the receptors VEGFR-1 (or Flt-1) and VEGFR-2. In PE this increases the release of FMS-like tyrosine kinase-1 (sFlt-1) and blocks free VEGF to protect the fetus from toxicity (136).
4. Neutrophils (PMN) are regulated by cytokines and prostaglandins (137). The aspirin (ASA) is used for prevention of preeclampsia in high-risk patients (138, 139). ASA triggers transcellular biosynthesis of eicosanoids by acetylation of PGHS-2. Eicosanoids correspond to 15R-epimers of lipoxins (ATL) and are potent inhibitors of leukotriene B4-mediated neutrophils (140). Considering that preeclampsia is associated with increased proinflammatory, antiangiogenic and PMN-endothelial cell adhesion, Gil-Villa et al. (141) shows that PMN adhesion in patients with preeclampsia is reduced by Aspirin-triggered lipoxin (ATL) when aspirin is used.
5. Natural killer and innate lymphoid cells (ILC). According to the cytokine profile and transcription factor, ILCs are divided into two groups, cytotoxic and “helper”-ILC (17). The cytotoxic ILC group is represented by Natural Killer (NK). The “helper”-ILC in humans has three subclasses, ILC1 with two subsets, producing IFNγ; ILC2 produces IL-5, IL-13, and IL-4; and ILC3 releases IL-17 and IL-22. The NK cells in a decidua (dNK) microenvironment are around 50% to 70% of the total of lymphoid cells in decidual tissue. They have CD56^bright^ CD16^−^ KIR^+^ CD9^+,^ and activate the NK receptor phenotype, participate with cytokines, which mediate new vessel formation, aid in the renovation of existing tissues and placentation through the release of VEGF, stromal-derived factor-1 (SDF-1) and IFN-γ-inducing protein 10 (9). In stromal tissue, the decidual stromal cells (DSCs) participate in the induction of maternal tolerance, physically concur and have a regulatory mechanism in dNK, and CD14^+^ myelomonocytic cells, and induce regulatory Treg. Also, DSCs inhibit dendritic cells through prostaglandin E2 (PGE2) and Indoleamine 2,3-dioxygenase (IDO), this inhibition favors the maintenance of the pregnancy (18).

In the normal eutopic endometrium, the Mø2 together with the Tregs predominate, providing an anti-inflammatory environment for the implantation of the embryo, while in endometriosis, they can cause infertility. The Mø1 provide a pro-inflammatory environment which affects embryo implantation, the dendritic cells (DC) do not increase in endometrial tissue, also the Treg is dysregulated. Therefore, DC does not eliminate the cellular debris which could migrate to the peritoneal cavity and grow in ectopic sites, developing as endometriosis. On the other hand, Treg and NK have abnormal behavior, the first favors a pro-inflammatory state and the second is less cytotoxic which impacts embryo implantation (142). COX2 and PGE2 are related to the pathogenesis of endometriosis. A high level of COX-2 due to various factors such as estrogens, hypoxia and environmental pollutants could suppress apoptosis and increase cell proliferation through PGE2 and its receptors EP2, and EP4 in endometriosis (143). In addition, experimental studies with intralesional injections of ASA, in rabbits with peritoneal endometriosis, eliminate endometriotic lesions (144).

## Prostaglandins in Implantation and Maintenance of Gestation

The generation of prostaglandins and expression of receptors in a mouse uterus has demonstrated their importance during implantation and decidualization (145). In mice, PGE2 levels increase from the 2-cell embryo stage to the blastocyst, demonstrating the importance of PGE2 in early development (112). PGE2 also plays a significant role in peri-implantation in a mouse uterus through the expression of EP2 and EP4 receptors, which increase cAMP levels during the implantation and decidualization processes. EP4 induces the activation of VEGF (growth factor vascular endothelial), increasing vascular permeability of the endometrium (146), implantation and decidualization, together with PGF2 (132).

Inadequate production of prostaglandins in mice, and possibly in humans, may explain some cases of infertility (147). Low concentrations of PGE2, PGF and PGI2 cause failure in ovulation, fertilization, implantation, and decidualization (133). In mice, prostacyclin (PGI2) is the primary prostaglandin at the implantation site. It participates in implantation and decidualization through the peroxisome proliferator-activated receptor (PPAR-δ) and the RXRα signaling pathway in the uterus (148).

As an example, PGF2α is used in fertilization procedures, in addition to GnRH, to pre-synchronize ovulation before applying for a resynchronization program in cows in dairy herds with acceptable pregnancy outcomes (149).

## Prostaglandins in Maternal Immune Tolerance

**Figure 1:**
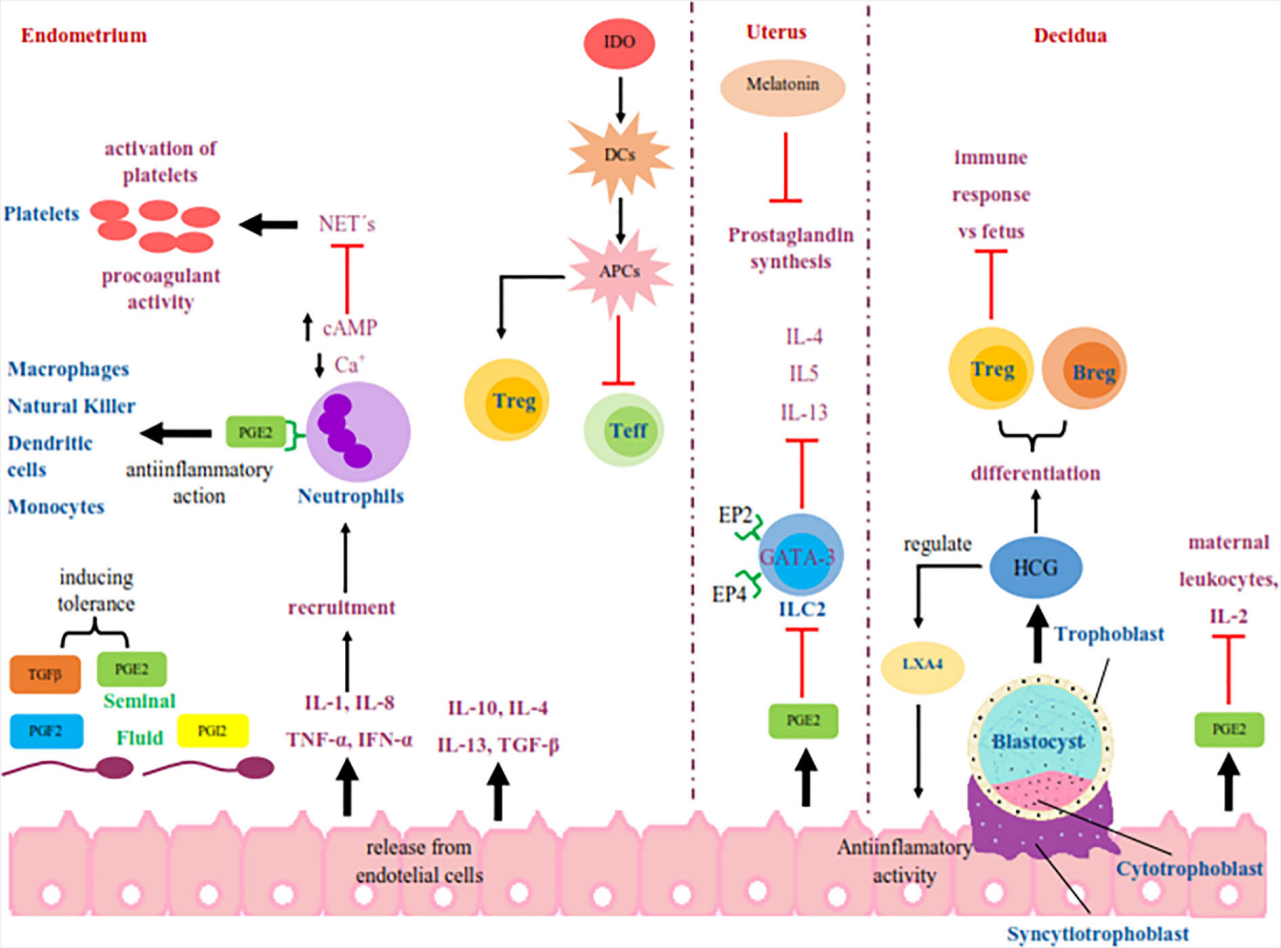
Schematic representation of the signaling in the maternal immune response that begins with the deposition of seminal fluid in the female reproductive tract during intercourse. The seminal fluid start an immune signaling pathways mediated by PGE2 and PGI2 in the functions of endothelial cells, platelets, neutrophils, ILC2, lymphocytes, macrophages, natural killer, dendritic cells and monocytes during oocyte fertilization and early implantation. In addition, the molecules released by these cells like interleukins, HCG, IDO, and LXA4 have a fundamental role in this tolerance process. PGE2, prostaglandin E2; PG12, prostaglandin I2; PGF2, prostaglandin F2; TGFβ, transforming growth factor beta; IL-1, interleukin-1; IL-2, interleukin-2; IL-4, interleukin-4; IL-5, interleukin-5; IL-8, interleukin-8; IL-10, interleukin-10; IL-13, interleukin-13; TNF-α, tumor necrosis factor-alpha; INF-α, interferón alpha; Ca+, calcio; cAMP, cyclic adenosine monophosphate; NET´s, neutrophil extracellular traps; IDO, indoleamine-2,3-dioxygenase; DCs, mature dendritic cells; APCs, tolerogenic antigen presenting cells; Treg, regulatory T cells; Teff, effector T cells; GATA-3, GATA-3 transcription factor; EP2, prostaglandin E2 receptor 2; EP4, prostaglandin E2 receptor 4; ILC2, group 2 innate lymphoid cells; Breg, regulatory B cells; HCG, human chorionic gonadotropin; LXA4, Lipoxin A4.

When intercourse occurs, endothelial cells release IL-8, IL-1, INF-α, and TNF-α to recruit immune cells (150). Neutrophils are mobilized in the oviduct in female mammals in response to the presence of sperm (151). This process may also induce a state of unresponsiveness by the presence of anti-inflammatory cytokines, such as IL-4, IL-10, IL-13, and TGF-β (152) **Figure 1**.

In order to prevent a compromised systemic maternal immune response, local immune regulation in the fetal-maternal interface is very important. This is achieved by several mechanisms. One of these is local immunoregulation at the fetal-maternal interface, e.g., Human amniotic membrane-derived mesenchymal stem cells (hAM-MSCs) release factors such as indoleamine 2,3 dioxygenase (IDO), TGF-β, prostaglandin E2 (PGE2), and others inducing immunomodulatory effects (153).

PGs release or regulate different kinds of cells, such as Tolerogenic dendritic cells (tol-DCs), Mø1 and Mø2 macrophages, Decidual NK cells (dNK) (CD56^bright^CD16^-^), Decidual stromal cells (DSCs), Endometrial stromal cells, Tregs (CD4^+^CD25^+^FOXP3^+^), and Decidual CD8^+^EM cells (CD45RA^−^CCR7^−^) (**Table 1**).

Prostaglandin E (PGE), specifically, induces T-helper type 3 (Th3) and T-regulatory 1 cells (Tr1), as shown by Lewis´ rat and mouse test (154, 155). PGE2 secretion by human deciduous cells in the first trimester of pregnancy blocks the activation of maternal leukocytes in the decidua and inhibits IL-2 production and its receptor (156).

Other cells assisting in the decidualization of endometrial stromal cells (ESCs) and pregnancy maintenance are decidual natural killer (dNK) cells (157) and CD14^+^ cells for Treg induction and immunosuppression (158). Also, Treg and Breg may contribute to the regulation of type 1 and 2-like T helper anti-fetal immune mechanisms during human pregnancy (159) (**Table 1**).

## Platelets

It is evident that platelets may be important in tolerance mechanisms. Platelet activity is inhibited post-coitus, and this inhibition depends on prostaglandins (160). Seminal fluid has factors that favor clot formation, similar to peripheral blood, such as Factor VIII: Ag, FVIII: C and Von Willebrand factor (vWF), in addition to other factors (161). vWF (162), fibronectin (163), and vitronectin (164) are proteins that favor platelet adhesion (165). This implies that inhibition of platelet aggregation by PGI2 could be a compensatory mechanism for pro-adhesive molecules.

Using a mouse model, Etulain et al. (166) found that platelets act through P-selectin glycoprotein ligand-1 (PSGL-1), and directly affect neutrophil extracellular traps (NETosis). Platelet P-selectin is crucial for neutrophil recruitment (167). Furthermore, NETs cause the recruitment and activation of platelets and induce procoagulant activity due to the expression of histones H3 and H4, toll-like receptor 2 (TLR2) and TLR4 platelets. NETs present a surface for the activation of coagulation factor XII (168) in order to promote thrombosis as a mechanism of rejection (169).

Platelets cause a decrease in the formation of extracellular traps when preincubated with PGI2, followed by stimulation with lipopolysaccharide (LPS), arachidonic acid, and a synthetic diacylated lipopeptide (Pam3SCK4). This highlights the physiological role of PGI2 in platelet modulation (170). Prostaglandins may also inhibit the function of neutrophils by increasing levels of cyclic adenosine monophosphate (cAMP) (171).

The interaction of PMN-platelets releases products of arachidonic acid serving as precursors of neutrophil eicosanoids (172). In polymorphonuclear neutrophils (PMN), PGE2 modulates their response through the expression of EP2 and EP4 receptors (173).

In addition, other mechanisms of maternal immune tolerance are mediated by placental trophoblast derived microvesicles (MVs) and maternal thrombocyte-derived MVs. These bind to circulating peripheral T lymphocytes through P-selectin (CD62P)–PSGL-1 (CD162) interaction induces STAT3 phosphorylation in T cells (174).

The above mentioned may explain why platelet aggregation is inhibited post-intercourse and has a possible reduction in the formation of NETs to protect the embryo. It is possible that the release of extracellular traps may contribute to trophoblast lesions.

Many other cells mentioned above participate through high complexity fetal-maternal interface interaction to induce a tolerance stage, which protects the embryo (175).

## Polymorphonuclear Cells

In mammalian species, PMNs are implicated in endometrial remodeling as being receptive to oocyte implantation. Human neutrophils exposed to progesterone and estriol hormones promote the establishment of maternal tolerance through the induction of CD4+ T cells (176).

In humans, during coitus, sperm is deposited into the female reproductive tract (FRT). Neutrophils are then recruited for the elimination of excess sperm through phagocytosis (177).

However, bovine seminal plasma is shown to reduce the ability of PMNs to phagocytize bull sperm. Furthermore, equine seminal plasma is reported to contain factors that reduce the binding of neutrophils to sperm, avoiding the formation of NETs (178). In humans, when granulocytes are exposed to the seminal plasma, the respiratory burst is inhibited (179). These mechanisms allow more of the healthy motile sperm to reach the oviduct, which makes it clear that seminal plasma contains factors that modulate the response of PMN.

In addition, PGE2 can exert anti-inflammatory action on neutrophils and other innate immune cells such as macrophages, natural killer cells, dendritic cells, and monocytes (180, 181). Also, it inhibits the production of IFN-α in plasmacytoid dendritic cells and the production of IL-12 in myeloid dendritic cells.

Finally, polymorphonuclear leukocytes contribute to preterm labor by activating prostaglandin production from human fetal membranes (182).

## Group 2 Innate Lymphoid Cells

Specific ILC2s (Group 2 innate lymphoid cells) and uterine innate lymphoid cells (uILCs, uILC1, uILC2, and uILC3) (183) in the uterus are regulated by PGD2, PGE2, PGI2, and sex hormones, in particular, oestrogen (151, 184). Together, these may play a role in the balance between immunity and tolerance at the beginning of placenta formation and could be related to pregnancy loss, as shown in mice (185). Some studies show that ILC2 is the most abundant subset in the human fetal-maternal interface during premature and full-term pregnancies, in which its presence is regulated by sex hormones (e.g., oestrogen) (186). PGI2 decreases the proliferation of ILC2 and significantly inhibits the expression of IL-5 and IL-13 induced by IL-33 (187).

The production of PGE2 could also suppress the function of neutrophils and uILCs, a particular cell, similar to ILC2, through its EP2 and EP4 receptors in both healthy humans and mouse models (188, 189). PGE2 inhibits the expression of GATA-3, as well as the production of type 2 cytokines (IL-5 and IL-13) (144). These effects are mediated by the action of the EP2 and EP4 prostanoid receptors, which are specifically expressed in ILC2 (151, 190).

In addition, Group 1ILCs, uNK cells, and uILC3s significantly increase in abortion in mice. They also have a lower proportion of uILC2s (183).

## Discussion

Of the hundreds of molecules released with cells in the preimplantation, implantation, and decidualization processes; prostaglandins are integrated into each of these stages by seminal fluid, even until parturition. In particular, some of these molecules are found to be related to infertility and abortions, such as PGE2, PGF, and PGI2, which, in turn, are related to ovulation, fertilization, implantation, and decidualization (133). Increased levels of IL6 are also related to unexplained infertility, recurrent miscarriage, and pre-eclampsia among other disorders (9), e.g., in humans, cases of placental insufficiency, manifesting as intrauterine fetal growth restriction, are observed where the level of melatonin, a molecule with pleiotropic effects that regulates inflammatory processes (191), is decreased (192). Melatonin inhibits prostaglandin synthesis and is a potent inducer of uterine contractility (54, 193), in addition, there is evidence that in fish, melatonin is produced in the granulosa cells and is a critical factor for ovulation (194). Likewise, in women, it increases progesterone and regulates the corpus luteum (195). Also in a recent clinical trial, melatonin is shown to improve intrafollicular oxidative balance and gives a slight increase in the rate of human live births (196). Another example is Polish landrace gilts treated with pregnant mare serum gonadotropin (PMSG) and human chorionic gonadotropin (hCG) (PMSG/hCG-induced). Treatment with exogenous progesterone increases pregnancy success through the expression of genes responsible for vascular function and PGE2 synthesis (197). Therefore, the administration of inhibitors of prostaglandin synthesis, e.g., PGE2, must be carefully considered due to the multiple mechanisms of female fertility in which they participate (111).

Also, the mechanism of control over the rate of gene transcription or transcriptional regulation is altered in genes involved in chronic endometritis and the inflammatory response (IL-11, CCL4), growth factors (IGFBP1), and apoptotic proteins (BCL2, BAX, CASP8) in infertile patients (198).

Another mechanism of transcriptional regulation is that of Uterine Vascular Endothelial Growth Factor (UVEGF), in which PGE2 regulates vascular development through receptors EP2 and EP4.

## Conclusions

To maintain fetal-maternal tolerance in the process of implantation (apposition/adhesion/invasion), a whole network of cells and molecules regulate different factors and responses according to the stage of pregnancy. Among the most highly studied cells and molecules are tolerogenic dendritic cells (tol-DCs), M1 and M2 macrophages, Decidual NK cells (dNK) (CD56brightCD16^−^), Decidual stromal cells (DSCs), Endometrial stromal cells, Tregs (CD4^+^ CD25^+^ FOXP3^+^) and Decidual CD8^+^ EM cells (CD45RA^−^ CCR7^−^), progesterone, oestrogen, Leukaemia inhibitory factor (LIF), Indoleamine-2,3-dioxygenase (IDO), and melatonin. Within this complex network, prostaglandins, specifically, PGD2, PGF2α, and PGE2, are important modulators and regulators in maintaining maternal-fetal tolerance, as we deduced. Nevertheless, other cells such as platelets, uILCs, and polymorphonuclear leukocyte/Nets require more research.

## Author Contributions

Conceptualization: EP-C and GM. Writing—original draft preparation: GM, GV, LP-C, MH-H, EC-P. Manuscript revision: GM, LP-C, MH-H, EZ, EP-CM, MM, RM, CM-C, NM, CR, EC-P, and EP-C. All authors contributed to the article and approved the submitted version.

## Funding

This research was supported by from National Technological of Mexico/ITOaxaca (project 5302.19-P) and Benito Juárez Autonomous University of Oaxaca (UABJO-CA-056). This work was supported by the Clinical Pathology Laboratory “Dr Eduardo Perez Ortega” in Oaxaca, Mexico.

## Conflict of Interest

The authors declare that the research was conducted in the absence of any commercial or financial relationships that could be construed as a potential conflict of interest.
